# Association between circulating GTP cyclohydrolase 1 concentrations and acute ischemic stroke: an exploratory case–control study in a Chinese population

**DOI:** 10.3389/fneur.2026.1842462

**Published:** 2026-08-18

**Authors:** Shengshan Yuan, Chen Yang, Phaik Har Yong, Zhi Xiang Ng, Baoyan Ren, Miao Li, Guijiang Wei, Lina Liang

**Affiliations:** 1 Department of Neurology, Affiliated Hospital of Youjiang Medical University for Nationalities, Baise, Guangxi, China; 2 School of Bioscience, Faculty of Pharmacy and Biomedical Sciences, MAHSA University, Selangor, Malaysia; 3 Baise Key Laboratory of In Vitro Diagnostic Technology, Baise, Guangxi, China; 4 Guangxi Key Laboratory of Artificial Intelligence for Genetic Diseases of Long-dwelling Nationalities, Baise, Guangxi, China; 5 Engineering Research Center of Guangxi Higher Education Institutions for Precise Genetic Testing of Long-dwelling Nationalities, Baise, Guangxi, China; 6 Key Laboratory of Research on Clinical Molecular Diagnosis for High Incidence Diseases in Western Guangxi of Guangxi Higher Education Institutions, Baise, Guangxi, China; 7 School of Biological and Environmental Sciences, Faculty of Science and Engineering, University of Nottingham Malaysia, Selangor, Malaysia; 8 Yaneng BlOscience (Shenzhen) Corporation, Shenzhen, Guangdong, China; 9 Clinical Genome Center, Guangxi KingMed Diagnostics, Nanning, Guangxi, China

**Keywords:** acute ischemic stroke, biomarker, enzyme-linked immunosorbent assay, GTP cyclohydrolase 1, tetrahydrobiopterin

## Abstract

**Background and objective:**

Ischemic stroke (IS) is a leading global cause of disability and mortality, characterized by cerebral hypoxia and tissue necrosis. GTP cyclohydrolase 1 (GCH1) regulates endothelial function and oxidative stress; however, whether circulating GCH1 concentrations are altered in acute ischemic stroke (AIS) remains unclear. This exploratory case–control study aimed to investigate plasma GCH1 levels and their associations with clinical characteristics in patients with AIS.

**Methods:**

Seventy-one patients with AIS and 92 controls undergoing routine health examinations were recruited at the Affiliated Hospital of Youjiang Medical University for Nationalities (January 2024–May 2025). Clinical and biochemical data including lipid profiles, C-reactive protein, homocysteine, and National Institutes of Health Stroke Scale (NIHSS) scores (only for patients with AIS) were collected. Plasma GCH1 levels were measured using an enzyme-linked immunosorbent assay. Statistical analyses were performed to evaluate differences between groups and to examine the associations between plasma GCH1 levels and clinical characteristics. Receiver operating characteristic curve analysis was conducted to assess the discriminatory performance of circulating GCH1.

**Results:**

Plasma GCH1 concentrations were significantly lower in AIS patients (6.51 ± 3.59 ng/mL vs. 14.32 ± 3.29 ng/mL, *p* < 0.001). Binary logistic regression analysis showed that lower plasma GCH1 levels were independently associated with AIS (OR = 0.496, 95% CI: 0.385–0.644, *p* < 0.001), while multiple linear regression analysis demonstrated that AIS was independently associated with lower plasma GCH1 levels (*B* = −7.687, 95% CI: −9.011 to −6.362, *p* < 0.001). Plasma GCH1 showed strong discrimination between the two groups (AUC = 0.924, 95% CI: 0.871–0.978) but was not associated with NIHSS scores (Spearman’s rho = −0.034, *p* = 0.778).

**Conclusion:**

Plasma GCH1 concentrations were lower in patients with AIS than in health-examination controls and showed high apparent discrimination in this dataset. Because GCH1 was measured after stroke onset and the sample-derived threshold was derived in the same case–control sample, these findings do not establish temporality, causality, or clinical diagnostic utility. Prospective multicenter studies including clinically relevant disease controls and independent external validation are required.

## 1 Introduction

Ischemic Stroke stands as one of the leading global causes of disability and mortality, characterized by persistently high incidence, disability, and recurrence rates (1, 2). Findings from China’s third national cause of death sampling survey indicate that stroke has become the primary cause of death in China (3). In Martin J. O′Donnell’s study conducted across 32 countries, among 13,447 cases of acute stroke, 10,388 were ischemic strokes (IS), accounting for 77.25% of the total (4). According to the Global Stroke Fact Sheet 2025, IS accounts for 65.3% of all stroke cases worldwide (1). Across 204 countries and territories, it accounted for approximately 50.5% of stroke-related deaths, translating to an estimated 3.6 million deaths in 2021 (5). This places a substantial healthcare and economic burden on families and societies alike. The pathophysiological process of AIS is complex; its core mechanism involves inadequate blood perfusion to local brain tissue due to vascular occlusion. This, in turn, triggers a cascade of events including hypoxia, enhanced oxidative stress, endothelial dysfunction, and activation of inflammatory responses, ultimately leading to neuronal necrosis and neurological deficits (6–8). Although significant progress has been made in recent years in treatment technologies such as thrombolysis and thrombectomy (9–12), the acute onset of AIS and the lack of specific biomarkers for early diagnosis often leads to delayed treatment and suboptimal outcomes for some patients. Consequently, identifying biomarkers that reflect the underlying pathophysiological processes of AIS may provide complementary information regarding disease severity, vascular injury, oxidative stress status, and potential therapeutic targets. Such biomarkers may enhance biological characterization and risk stratification of AIS when used alongside established clinical and neuroimaging assessments (13, 14).

GTP cyclohydrolase 1 (GCH1) is the rate-limiting enzyme in the biosynthesis of tetrahydrobiopterin (BH4) *in vivo*. As a key cofactor for nitric oxide synthase (NOS), BH4 regulates vascular endothelial function by maintaining the active conformation of NOS and also scavenges reactive oxygen species (ROS) to suppress oxidative stress, thereby playing a central role in the maintenance of vascular homeostasis (15, 16). Extensive experimental evidence has demonstrated that GCH1 regulates BH4 biosynthesis, endothelial nitric oxide synthase coupling, nitric oxide bioavailability, and oxidative stress responses (16–18). Furthermore, GCH1 deficiency has been implicated in endothelial dysfunction and atherosclerotic progression in both cellular and animal studies (19, 20). These findings support a biologically plausible link between GCH1 and vascular diseases. However, whether circulating GCH1 concentrations are altered in patients with AIS and whether such alterations are associated with clinical characteristics, inflammatory status, or neurological impairment remain largely unknown. Therefore, the present study was designed to explore these potential associations rather than to establish a causal role of GCH1 in AIS pathogenesis.

Based on the aforementioned research background, this study employed a case–control design to quantify plasma GCH1 levels in patients with AIS and in healthy control subjects. The associations between plasma GCH1 levels and clinical characteristics, including sex, age, blood lipid profiles, C-reactive protein (CRP), homocysteine concentrations, and National Institutes of Health Stroke Scale (NIHSS) scores are systematically evaluated. Furthermore, the potential role of GCH1 as an independent factor associated with AIS is investigated. This study aims to provide preliminary evidence of the association between circulating GCH1 levels and AIS, thereby improving our understanding of the potential role of the GCH1–BH4 pathway in vascular dysfunction and oxidative stress in the context of ischemic stroke. In addition, the findings may help assess the potential utility of GCH1 as a biomarker of pathophysiological processes in AIS.

## 2 Materials and methods

### 2.1 Ethical approval and study participants

**Figure 1:**
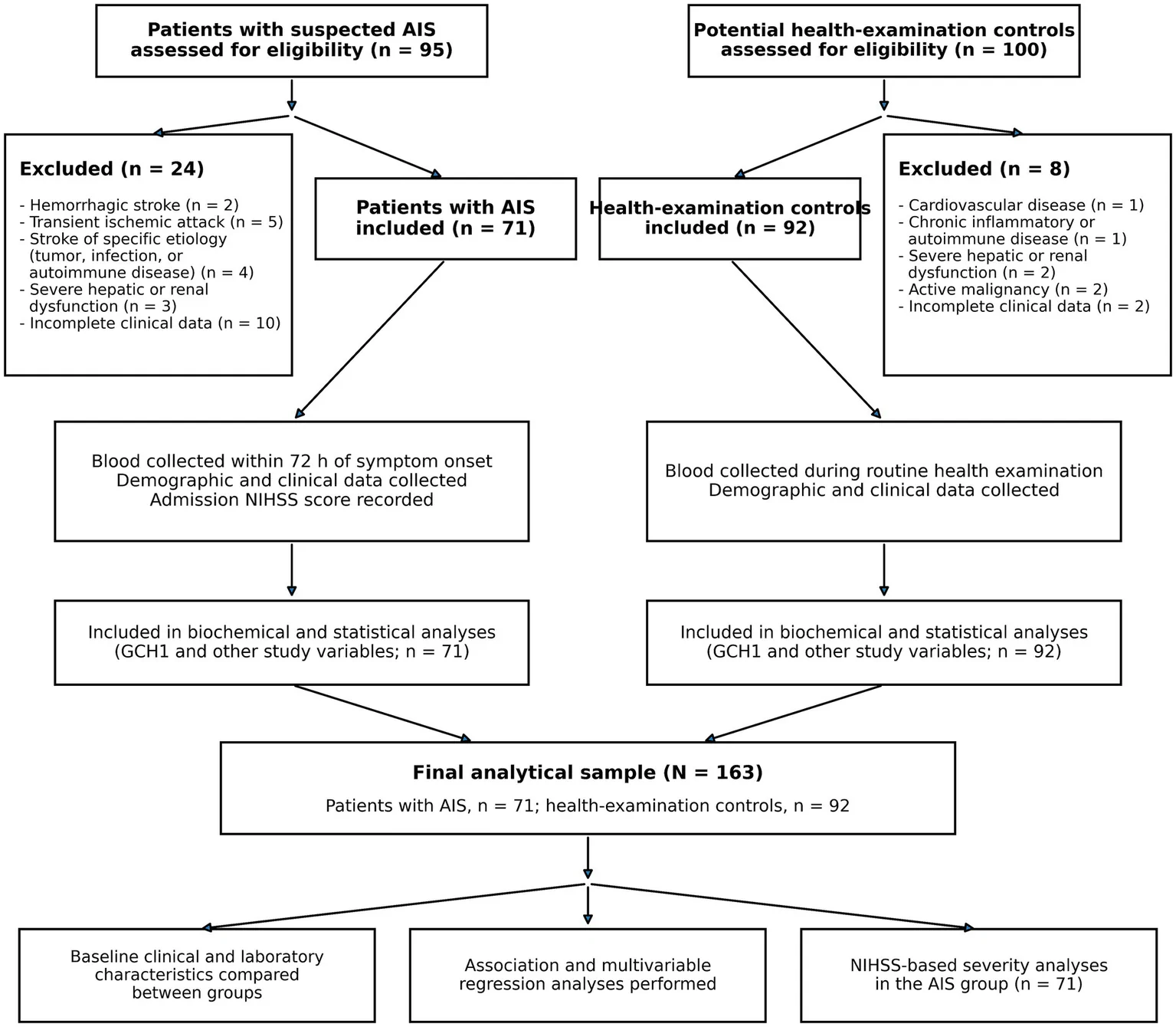
Participant flow diagram. A total of 95 patients with suspected acute ischemic stroke (AIS) were assessed for eligibility. After the exclusion of 24 patients, 71 patients with AIS were included. Among 100 potential controls undergoing routine health examinations, 8 were excluded and 92 were included. The final analytical sample comprised 163 participants, including 71 patients with AIS and 92 health-examination controls. All participants provided written informed consent before enrollment. AIS, Acute ischemic stroke; GCH1, GTP cyclohydrolase 1; NIHSS, National Institutes of Health Stroke Scale.

This study was approved by the Ethics Committee of the Affiliated Hospital of Youjiang Medical University for Nationalities (Approval No. 2022112301), and written informed consent was obtained from all participants prior to enrollment. Adults aged ≥18 years were enrolled in the current study. The case group comprised patients diagnosed with acute cerebral infarction, defined by characteristic clinical manifestations and neuroimaging findings in accordance with the Chinese Guidelines for the Diagnosis and Treatment of Acute Ischemic Stroke (21), with symptom onset within 72 h. The control group consisted of health-examination controls without AIS or previous stroke undergoing routine health examinations during the same study period. No individual matching procedure was performed; controls were recruited independently of case participants. General demographic and clinical data were collected by trained professionals from the Department of Neurology and the Health Examination Center of the Affiliated Hospital of Youjiang Medical University for Nationalities. Collected variables included sex, age, history of hypertension and diabetes, smoking status, and NIHSS score. Admission NIHSS scores were assessed only in patients with AIS by trained neurologists. Participants were excluded if they had a history of previous cardiovascular or cerebrovascular disease, heart failure, hepatic or renal failure, severe pulmonary infection, pregnancy, malignancy, hematological disorders, recent major surgery or trauma, autoimmune diseases, chronic inflammatory diseases, or other severe systemic conditions. The sample size was estimated using the formula for comparing two independent means: *n* = 2(Z_*α*/2_ + Z*_β_*)^2^ / *d^2^*. Where *d* = *δ*/*σ* represents Cohen’s standardized effect size. Based on Cohen’s criteria, a medium effect size (*d* = 0.5) was assumed. Using a two-sided significance level of α = 0.05 and a statistical power of 80% (*β* = 0.20), the minimum required sample size was calculated to be 63 participants per group. Following application of the inclusion and exclusion criteria, a total of 71 patients with acute cerebral infarction admitted to the Department of Neurology between January 2024 and May 2025 and 92 health-examination controls recruited during the same period were included in the final analysis, exceeding the calculated minimum sample size requirement. The participant flow diagram illustrating participant selection is shown in Figure 1, and the clinical characteristics of all participants are summarized in Table 1.

**Table 1:** Comparison of clinical characteristics between the case group and control group.

Variable	Case group (*n* = 71)	Control group (*n* = 92)	*p*-value
Age (years, mean ± SD)	62.06 ± 10.59	54.29 ± 8.26	<0.001
Men (*n*, %)	48(67.6%)	46 (50.0%)	0.024
Hypertension (*n*, %)	55 (77.5%)	26 (28.3%)	<0.001
Diabetes (*n*, %)	21 (29.6%)	16 (17.4%)	0.066
Smoking (*n*, %)	21 (29.6%)	5(5.4%)	<0.01
TG (mmol/L)	1.50 ± 0.97	1.62 ± 1.25	0.506
TC (mmol/L)	4.61 ± 1.17	5.15 ± 1.23	0.005
HDL-C (mmol/L)	1.07 ± 0.33	1.39 ± 0.54	<0.001
LDL-C (mmol/L)	2.85 ± 0.99	3.25 ± 1.02	0.013
APOA1 (g/L)	1.49 ± 0.42	2.04 ± 0.51	<0.001
APOB (g/L)	0.99 ± 0.29	1.10 ± 0.33	0.026
Lp(a) (nmol/L)	60.04 ± 73.55	37.11 ± 56.74	0.032
HCY (umol/L)	12.83 ± 6.79	12.89 ± 5.58	0.950
CRP (mg/L)	17.07 ± 34.57	4.35 ± 4.29	0.003
GCH1 (ng/ml)	6.51 ± 3.59	14.32 ± 3.29	<0.001

Continuous variables are presented as mean ± standard deviation (SD) and compared using the independent-samples *t*-test or Mann–Whitney *U*-test as appropriate. Categorical variables were compared using the *χ*^2^ test or Fisher’s exact test. triglyceride (TG); total cholesterol (TC); high-density lipoprotein cholesterol (HDL-C); low-density lipoprotein cholesterol (LDL-C); apolipoprotein A1 (APOA1); apolipoprotein B (APOB); lipoprotein(a) [Lp(a)]; homocysteine (HCY); C-reactive protein (CRP); GTP cyclohydrolase 1 (GCH1).

### 2.2 Sample collection

Ten millilitres (10 mL) of venous blood were collected from patients with AIS between 6:00 and 7:30 a.m. on the day of admission or the following morning after an overnight fast. Consequently, the interval between symptom onset and blood collection varied among patients; however, all blood samples were collected within 72 h of symptom onset. For health-examination control participants, venous blood samples were collected between 7:30 and 9:00 a.m. after an overnight fast using the same protocol. Blood samples were collected into EDTA-containing vacuum tubes. All samples were centrifuged at 3,000 × g for 10 min at 4 °C to separate plasma, which was then aliquoted and stored at −80 °C until analysis. All samples were processed, aliquoted, and stored according to the same standardized protocol under identical conditions. No repeated freeze–thaw cycles occurred before analysis, and there was no systematic difference in storage duration between the case and control groups. To minimize inter-assay variability, all plasma samples were analyzed simultaneously in a single batch after completion of sample collection.

### 2.3 Laboratory blood parameter analyses

Plasma levels of total cholesterol (TC), triglyceride (TG), high-density lipoprotein cholesterol (HDL-C), low-density lipoprotein cholesterol (LDL-C), apolipoprotein A1 (APOA1), apolipoprotein B (APOB), lipoprotein(a) [Lp(a)], C-reactive protein (CRP), and homocysteine (HCY), were measured using a fully automated biochemical analyzer (Roche, Germany; Model: cobas 8,000 C702) in the hospital’s clinical central laboratory. Data were retrieved from the hospital information system.

### 2.4 Measurement of plasma GCH1

Plasma GCH1 concentrations were measured using a commercially available human GCH1 enzyme-linked immunosorbent assay (ELISA) kit (mlbio Enzyme-linked Biotechnology, ml061636). The assay had a quantitative range of 0.3125–10 ng/mL and a limit of detection of <0.1 ng/mL.

All ELISA procedures were performed in a blinded manner. Before laboratory analysis, plasma samples were anonymized by re-coding and randomized by an independent coordinator who was not involved in sample testing or outcome evaluation. Laboratory personnel and data analysts remained blinded to participants’ case–control status throughout the experimental and analytical processes until completion of data analysis and database lock.

Plasma samples were diluted 1:2 using the sample diluent supplied with the kit, which has a reported recovery rate of 98%, and all experimental procedures were performed in accordance with the manufacturer’s instructions. ELISA standard curves were generated using standards at concentrations of 10, 5, 2.5, 1.25, 0.625, and 0.3125 ng/mL, covering a quantitative range of 0.3125–10 ng/mL. Standard curves were fitted using a four-parameter logistic regression model. For each ELISA plate, assay acceptance criteria required a coefficient of determination (*R*^2^) ≥ 0.995 for the standard curve and a coefficient of variation (CV) < 5% for duplicate measurements at each standard concentration.

Absorbance was measured using a fully automated ELISA analyzer (Aikang Biotech, Shenzhen, China; URANUS AE275 A-6). Most samples yielded readings within the assay’s quantitative range after the initial 1:2 dilution. Nine samples with readings above the upper limit of the standard curve were re-assayed at higher serial dilutions until their measured values fell within the quantitative range. Final plasma concentrations were calculated by multiplying the measured values by the corresponding total dilution factor. Samples with diluted concentrations exceeding the upper limit of the standard curve (10 ng/mL) were subjected to additional serial dilutions (e.g., 1:4, 1:8, or higher) in accordance with the manufacturer’s instructions and reanalyzed until the measured values fell within the quantitative range. Final concentrations were calculated as the measured concentration multiplied by the total dilution factor.

All samples were analyzed in duplicate within a single assay batch, and the mean of the two measurements was used for subsequent analyses. The intra-assay and inter-assay coefficients of variation were <10 and <15%, respectively. Because all plasma samples were analyzed simultaneously in a single batch after collection, each sample underwent only one freeze–thaw cycle, thereby minimizing variability associated with repeated freeze–thaw cycles.

### 2.5 Statistical analysis

Normality of continuous variables was assessed using the Shapiro–Wilk test. Quantitative data conforming to a normal distribution were expressed as the mean ± standard deviation (X ± SD). Comparisons of quantitative data between two groups that exhibited a normal distribution and homogeneity of variance were performed using Student’s t-test. Because plasma GCH1 concentrations were not normally distributed across the NIHSS-defined severity groups, differences among patients with mild, moderate, and severe neurological deficits were evaluated using the Kruskal–Wallis test. Plasma GCH1 concentrations in these groups are presented as the median and interquartile range (IQR). For graphical presentation, box-and-whisker plots with individual observations overlaid were used; boxes represent the IQR, horizontal lines within the boxes indicate the medians, and whiskers extend to 1.5 × IQR. Data not following a normal distribution were analyzed using the Mann–Whitney *U*-test (rank-sum test), while differences in rates (or proportions) were assessed using Pearson’s chi-square test. Spearman correlation, binary logistic regression, and multiple linear regression were used to determine the association of different variables. A two-sided *p* value of < 0.05 was considered statistically significant. All experimental data were analyzed with statistical software packages GraphPad Prism 9.0 and SPSS 27.0.

## 3 Results

### 3.1 Baseline characteristics of the study participants

A total of 163 participants were included in this study: 71 patients constituted the AIS case group, and 92 health-examination controls comprised the control group. Baseline clinical characteristics and laboratory parameters for both groups are summarized in Table 1.

The data suggest that the case group was, on average, significantly older than the control group (62.06 ± 10.59 years vs. 54.29 ± 8.26 years; *p* < 0.001). In addition, the proportion of males was significantly higher in the case group than in the control group (67.6% vs. 50.0%, *p* = 0.024). Furthermore, the case group exhibited a substantially higher prevalence of established cardiovascular risk factors. The prevalence of hypertension was significantly higher in the case group compared with the control group (77.5% vs. 28.3%; *p* < 0.001). Similarly, a history of smoking was more frequently observed in the case group (29.6% vs. 5.4%, *p* < 0.01). The case group also had a higher prevalence of diabetes (29.6% vs. 17.4%), but this difference did not reach conventional statistical significance (*p* = 0.066).

The lipid panel revealed a complex profile. The case group had statistically significantly lower levels of TC, LDL-C, and APOB. Both HDL-C and its primary structural protein, APOA1, were significantly lower in the case group (*p* < 0.001 for both). Conversely, the case group had a significantly elevated level of CRP (17.07 mg/L vs. 4.35 mg/L; *p* = 0.003). Lp(a), which is an independent genetic risk factor for atherothrombosis, was also significantly higher in the case group (*p* = 0.032). Plasma GCH1 concentrations ranged from 3.61 to 24.29 ng/mL. Mean plasma GCH1 levels were significantly lower in the case group than in the control group (6.51 ± 3.59 vs. 14.32 ± 3.29 ng/mL, *p* < 0.001), representing a reduction of approximately 55%. No statistically significant differences were observed between the groups in terms of TG levels, or HCY levels.

### 3.2 Influencing factors analysis in AIS

As shown in Table 1, significant differences were observed between the case and control groups in terms of age, sex, hypertension, smoking, and plasma levels of TC, HDL-C, LDL-C, APOA1, APOB, Lp(a), CRP, and GCH1. To clarify the impact of these indicators on AIS, a binary logistic regression model was performed.

Considering both clinical relevance and the statistically significant differences shown in Table 1, candidate variables were selected for multivariable analysis. After assessment of multicollinearity, sex, hypertension, diabetes, smoking status, age, HDL-C, LDL-C, Lp(a), CRP, and GCH1 were entered into a binary logistic regression model to identify variables independently associated with AIS.

The overall logistic regression model was statistically significant, with a likelihood ratio *χ*^2^ of 166.930 and 10 degrees of freedom (*p* < 0.001). The model showed good explanatory performance, with a McFadden pseudo *R*^2^ of 0.748 and a Nagelkerke *R*^2^ of 0.859. The area under the receiver operating characteristic (ROC) curve was 0.978, indicating excellent discriminative ability (Table 2).

**Table 2:** Model fit and apparent model discrimination of the multivariable logistic regression model for AIS.

Indicator	Value
Log-likelihood	−28.161
Null log-likelihood	−111.626
−2 log-likelihood	56.323
Likelihood ratio *χ*^2^	166.930
Degrees of freedom	10
Likelihood ratio *p*-value	<0.001
McFadden pseudo *R*^2^	0.748
Cox–Snell *R*^2^	0.641
Nagelkerke *R*^2^	0.859
Akaike information criterion	78.323
Bayesian information criterion	112.354
Hosmer–Lemeshow *χ*^2^	13.200
Hosmer–Lemeshow degrees of freedom	8
Hosmer–Lemeshow *p*-value	0.105
Area under the ROC curve	0.978

The analysis revealed that Lp(a) (OR = 1.020, 95% CI: 1.006–1.034, *p* = 0.005) and hypertension (OR = 19.893, 95% CI: 2.749–143.961, *p* = 0.003) were independently associated with higher odds of AIS. In contrast, HDL-C (OR = 0.055, 95% CI: 0.004–0.868, *p* = 0.039) and GCH1 (OR = 0.496, 95% CI: 0.385–0.644, *p* < 0.001) were inversely associated with AIS. Smoking status, LDL-C, and CRP were not independently associated with AIS in the multivariable model (all *p* > 0.05; Table 3).

**Table 3:** Binary logistic regression analysis of factors with significant differences in AIS.

Independent variable	*B*	S.E.	Wald	*p*-value	OR	95%CI	
						Lower bound	Upper bound
Sex	0.273	0.977	0.078	0.780	1.314	0.194	8.911
Smoking	−0.021	0.969	<0.001	0.983	0.979	0.147	6.537
Hypertension	2.990	1.010	8.770	0.003	19.893	2.749	143.961
Diabetes	0.348	0.855	0.166	0.684	1.417	0.265	7.573
Age	−0.040	0.050	0.636	0.425	0.961	0.872	1.060
HDL-C	−2.898	1.406	4.245	0.039	0.055	0.004	0.868
LDL-C	0.474	0.379	1.564	0.211	1.606	0.764	3.375
Lp(a)	0.020	0.007	8.360	0.005	1.020	1.006	1.034
CRP	0.135	0.073	3.447	0.063	1.144	0.993	1.319
GCH1	−0.700	0.129	29.327	<0.001	0.496	0.385	0.644
Constant	11.300	4.663	5.872	0.015	80781.571		

Abbreviations for biochemical variables: HDL-C, high-density lipoprotein cholesterol; LDL-C, low-density lipoprotein cholesterol; Lp(a), lipoprotein(a); CRP, C-reactive protein; GCH1, GTP cyclohydrolase 1. Abbreviations for statistical parameters: B, partial regression coefficient; S.E., standard error; Wald, Wald chi-square statistic; df, degrees of freedom; OR, odds ratio.

### 3.3 Associations of plasma GCH1 with clinical and laboratory factors

To clarify the associations between plasma GCH1 levels and clinical or laboratory indicators, including age, smoking status, hypertension, TC, HDL-C, LDL-C, APOA1, APOB, Lp(a), CRP, and NIHSS score, stratified pairwise comparison and correlation analyses were performed within the AIS group, within the control group, and in the overall study population. Normality of continuous variables was assessed using the Shapiro–Wilk test, which showed that most variables were not normally distributed. Therefore, comparisons of plasma GCH1 levels across categorical variables, such as smoking status and hypertension, were performed using the Mann–Whitney *U*-test. Associations between plasma GCH1 levels and continuous variables, including age and lipid parameters, were evaluated using Spearman correlation analysis. To account for multiple testing, the Benjamini–Hochberg false discovery rate (FDR) correction was applied, and FDR-adjusted q values were reported.

In the case subgroup, no significant associations were observed between plasma GCH1 levels and either categorical or continuous clinical/laboratory variables after Benjamini–Hochberg FDR correction. Although hypertension and HCY showed nominal significance in the case subgroup, these associations did not remain significant after correction for multiple testing (Table 4).

**Table 4:** Associations of plasma GCH1 with clinical and laboratory indicators in the case subgroup.

Variable	N (0/1)	Test	Effect	95% CI	Statistic	*p*	FDR q
Sex	71 (23/48)	Mann–Whitney *U*-test	HL diff = 0.175	−0.449 to 0.750	*U* = 603.5	0.531	0.703
Smoking	71 (50/21)	Mann–Whitney *U*-test	HL diff = −0.018	−0.632 to 0.722	*U* = 517.5	0.930	0.943
Hypertension	71 (16/55)	Mann–Whitney *U*-test	HL diff = 0.706	0.192 to 1.313	*U* = 615.0	0.016	0.057
Diabetes	71 (50/21)	Mann–Whitney *U*-test	HL diff = 0.249	−0.408 to 1.038	*U* = 584.5	0.457	0.664
Age	71	Spearman correlation	rho = 0.104	−0.131 to 0.335	rho = 0.104	0.388	0.612
TG	71	Spearman correlation	rho = 0.155	−0.064 to 0.371	rho = 0.155	0.197	0.402
TC	71	Spearman correlation	rho = 0.081	−0.171 to 0.320	rho = 0.081	0.501	0.683
HDL-C	71	Spearman correlation	rho = −0.108	−0.328 to 0.140	rho = −0.108	0.369	0.612
LDL-C	71	Spearman correlation	rho = 0.048	−0.202 to 0.303	rho = 0.048	0.689	0.810
APOA1	71	Spearman correlation	rho = −0.020	−0.258 to 0.229	rho = −0.020	0.866	0.906
APOB	71	Spearman correlation	rho = 0.166	−0.093 to 0.403	rho = 0.166	0.166	0.374
Lp(a)	71	Spearman correlation	rho = 0.113	−0.126 to 0.344	rho = 0.113	0.346	0.599
CRP	71	Spearman correlation	rho = 0.044	−0.182 to 0.274	rho = 0.044	0.714	0.810
HCY	71	Spearman correlation	rho = 0.271	0.037 to 0.476	rho = 0.271	0.022	0.071
NIHSS	71	Spearman correlation	rho = −0.034	−0.288 to 0.207	rho = −0.034	0.778	0.833

In the Sex variable, 1 indicates male and 0 indicates female. For smoking, hypertension, and diabetes, 1 indicates the presence of a relevant history, whereas 0 indicates its absence. Categorical comparisons were performed using the Mann–Whitney *U*-test. For continuous variables, the effect column reports Spearman’s rho. The 95% CI for the HL difference and Spearman correlation coefficients were estimated using bootstrap resampling with 5,000 iterations. Multiple testing correction method: Benjamini–Hochberg FDR correction; the primary results are presented with global FDR correction applied to all valid tests. GCH1, GTP cyclohydrolase 1; HCY, homocysteine; Lp(a), lipoprotein(a); APOB, apolipoprotein B; APOA1, apolipoprotein A1; LDL-C, low-density lipoprotein cholesterol; HDL-C, high-density lipoprotein cholesterol; TC, total cholesterol; TG, triglyceride; NIHSS, National Institutes of Health Stroke Scale; CI, Confidence Interval; HL diff, Hodges–Lehmann difference; FDR, Benjamini–Hochberg false discovery rate.

In the control subgroup, smoking status was significantly associated with lower plasma GCH1 levels. The Hodges–Lehmann difference in GCH1 levels was −2.991, with a 95% confidence interval of −5.043 to −1.594, and this association remained significant after FDR correction (*p* = 0.006, FDR *q* = 0.026). In contrast, the associations of TC and LDL-C with plasma GCH1 levels reached only nominal significance and were no longer significant after FDR correction (Table 5).

**Table 5:** Associations of plasma GCH1 with clinical and laboratory indicators in the control subgroup.

Variable	N (0/1)	Test	Effect	95% CI	Statistic	*p*	FDR q
Sex	92 (46/46)	Mann–Whitney *U*-test	HL diff = −1.014	−2.259 to 0.031	*U* = 811.5	0.055	0.130
Smoking	92 (87/5)	Mann–Whitney *U*-test	HL diff = −2.991	−5.043 to −1.594	*U* = 57.0	0.006	0.026
Hypertension	92 (66/26)	Mann–Whitney *U*-test	HL diff = 0.286	−0.936 to 1.476	*U* = 909.0	0.661	0.810
Diabetes	92 (76/16)	Mann–Whitney *U*-test	HL diff = 0.258	−1.445 to 1.504	*U* = 641.0	0.738	0.810
Age	92	Spearman correlation	rho = −0.079	−0.284 to 0.140	rho = −0.079	0.456	0.664
TG	92	Spearman correlation	rho = 0.044	−0.143 to 0.239	rho = 0.044	0.674	0.810
TC	92	Spearman correlation	rho = 0.208	−0.015 to 0.409	rho = 0.208	0.047	0.119
HDL-C	92	Spearman correlation	rho = 0.075	−0.154 to 0.296	rho = 0.075	0.476	0.669
LDL-C	92	Spearman correlation	rho = 0.207	−0.018 to 0.401	rho = 0.207	0.048	0.119
APOA1	92	Spearman correlation	rho = 0.136	−0.083 to 0.346	rho = 0.136	0.196	0.402
APOB	92	Spearman correlation	rho = 0.132	−0.074 to 0.334	rho = 0.132	0.211	0.402
Lp(a)	92	Spearman correlation	rho = 0.008	−0.194 to 0.217	rho = 0.008	0.943	0.943
CRP	92	Spearman correlation	rho = −0.036	−0.250 to 0.181	rho = −0.036	0.733	0.810
HCY	92	Spearman correlation	rho = −0.106	−0.301 to 0.089	rho = −0.106	0.317	0.570

In the Sex variable, 1 indicates male and 0 indicates female. For smoking, hypertension, and diabetes, 1 indicates the presence of a relevant history, whereas 0 indicates its absence. Categorical comparisons were performed using the Mann–Whitney *U*-test. For continuous variables, the effect column reports Spearman’s rho. The 95% CI for the HL difference and Spearman correlation coefficients were estimated using bootstrap resampling with 5,000 iterations. Multiple testing correction method: Benjamini–Hochberg FDR correction; the primary results are presented with global FDR correction applied to all valid tests. Control subgroup NIHSS was not tested because all NIHSS values were 0 in that subgroup. Abbreviations for biochemical variables: GCH1, GTP cyclohydrolase 1; HCY, homocysteine; Lp(a), lipoprotein(a); APOB, apolipoprotein B; APOA1, apolipoprotein A1; LDL-C, low-density lipoprotein cholesterol; HDL-C, high-density lipoprotein cholesterol; TC, total cholesterol; TG, triglyceride; CI, Confidence Interval; HL diff, Hodges–Lehmann difference; FDR, Benjamini–Hochberg false discovery rate.

In the pooled analysis, plasma GCH1 concentrations were significantly associated with smoking status, hypertension, age, TC, HDL-C, LDL-C, APOA1, APOB, and Lp(a) after Benjamini–Hochberg FDR correction (all *q* < 0.05, Table 6). Sex and CRP showed nominal associations but did not remain significant after FDR correction. Because these pooled associations may have been driven by the marked separation between the AIS and control groups, they should be interpreted as supplementary exploratory findings.

**Table 6:** Overall population: Associations between plasma GCH1 and clinical/laboratory indicators.

Variable	N (0/1)	Test	Effect	95% CI	Statistic	*p*	FDR q
Sex	163 (69/94)	Mann–Whitney *U*-test	HL diff = −1.425	−3.713 to −0.176	*U* = 2569.0	0.024	0.071
Smoking	163 (137/26)	Mann–Whitney *U*-test	HL diff = −5.024	−7.164 to −2.036	*U* = 922.5	<0.001	0.001
Hypertension	163 (82/81)	Mann–Whitney *U*-test	HL diff = −4.367	−6.362 to −1.267	*U* = 2215.0	<0.001	0.002
Diabetes	163 (126/37)	Mann–Whitney *U*-test	HL diff = −0.589	−2.659 to 0.657	*U* = 2115.5	0.394	0.612
Age	163	Spearman correlation	rho = −0.284	−0.423 to −0.132	rho = −0.284	<0.001	0.002
TG	163	Spearman correlation	rho = 0.045	−0.096 to 0.190	rho = 0.045	0.569	0.731
TC	163	Spearman correlation	rho = 0.278	0.138 to 0.414	rho = 0.278	<0.001	0.002
HDL-C	163	Spearman correlation	rho = 0.233	0.084 to 0.377	rho = 0.233	0.003	0.014
LDL-C	163	Spearman correlation	rho = 0.258	0.112 to 0.399	rho = 0.258	<0.001	0.005
APOA1	163	Spearman correlation	rho = 0.405	0.262 to 0.534	rho = 0.405	<0.001	<0.001
APOB	163	Spearman correlation	rho = 0.205	0.057 to 0.345	rho = 0.205	0.009	0.035
Lp(a)	163	Spearman correlation	rho = −0.195	−0.339 to −0.045	rho = −0.195	0.012	0.047
CRP	163	Spearman correlation	rho = −0.157	−0.312 to 0.007	rho = −0.157	0.046	0.119
HCY	163	Spearman correlation	rho = 0.098	−0.064 to 0.247	rho = 0.098	0.214	0.402

In the Sex variable, 1 indicates male and 0 indicates female. For smoking, hypertension, and diabetes, 1 indicates the presence of a relevant history, whereas 0 indicates its absence. Categorical comparisons were performed using the Mann–Whitney *U*-test. For continuous variables, the effect column reports Spearman’s rho. The 95% CI for the HL difference and Spearman correlation coefficients were estimated using bootstrap resampling with 5,000 iterations. Multiple testing correction method: Benjamini–Hochberg FDR correction; the primary results are presented with global FDR correction applied to all valid tests. Abbreviations for biochemical variables: GCH1, GTP cyclohydrolase 1; HCY, homocysteine; Lp(a), lipoprotein(a); APOB, apolipoprotein B; APOA1, apolipoprotein A1; LDL-C, low-density lipoprotein cholesterol; HDL-C, high-density lipoprotein cholesterol; TC, total cholesterol; TG, triglyceride; CI, Confidence Interval; HL diff, Hodges–Lehmann difference; FDR, Benjamini–Hochberg false discovery rate.

**Figure 2:**
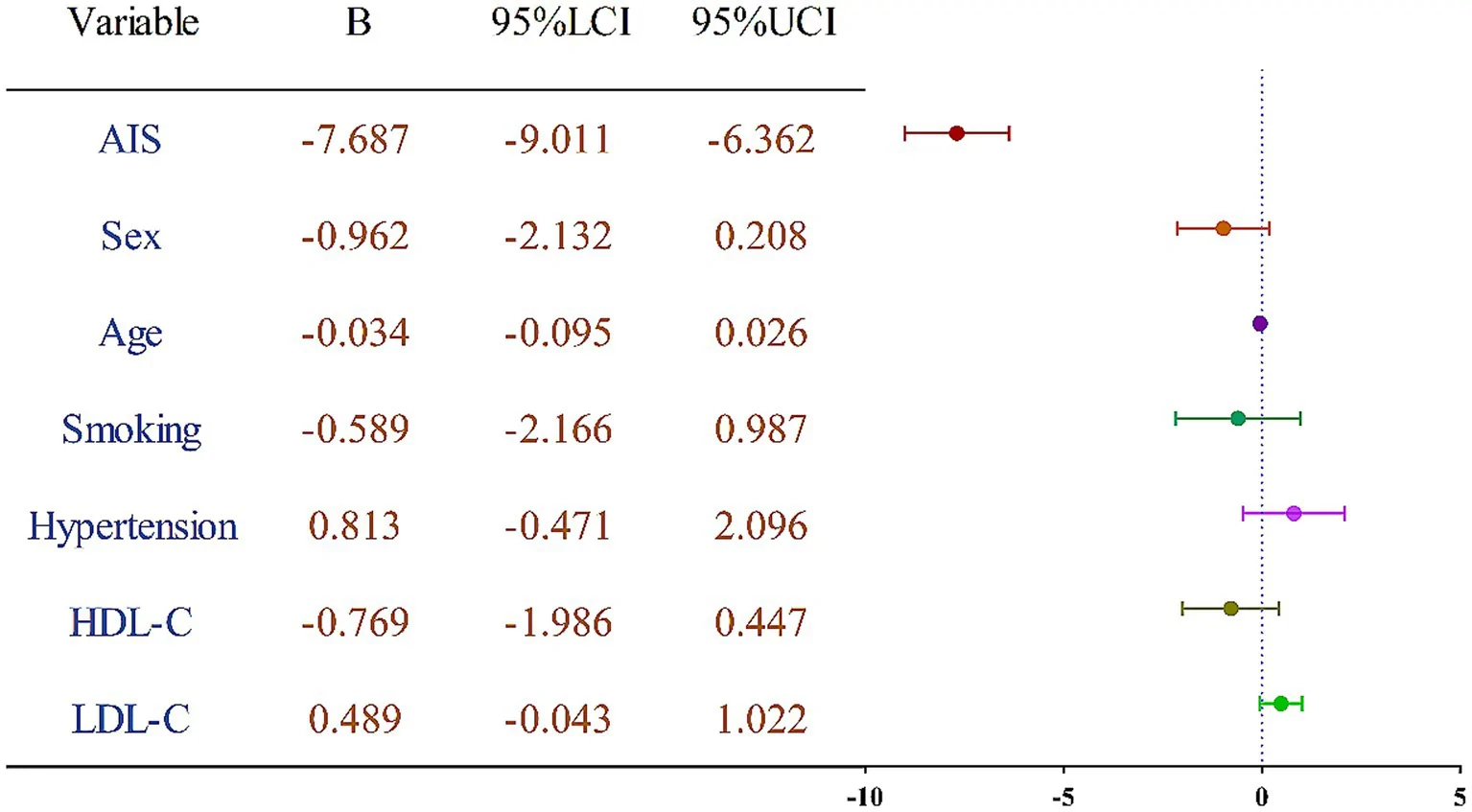
Forest plot of factors associated with plasma GCH1 levels. The horizontal axis represents the confidence interval, and the vertical axis represents each variable. Each line segment shows the upper and lower limits of the 95% confidence interval for each variable, with the midpoint corresponding to its *B* value. Results showed that after adjusting for other factors, AIS status was independently associated with an adjusted 7.687-ng/mL lower plasma GCH1 concentration (*B* = −7.687, 95% CI, −9.011 to −6.362; *p* < 0.001). Note: HDL-C, high-density lipoprotein cholesterol; LDL-C, low-density lipoprotein cholesterol; GCH1, GTP cyclohydrolase 1.

To identify independent factors associated with plasma GCH1 levels, a multiple linear regression analysis was conducted after confirming the absence of significant multicollinearity. Plasma GCH1 concentration was entered as the dependent variable, while AIS, sex, age, smoking status, hypertension, HDL-C, and LDL-C were included as independent variables. The analysis revealed that AIS was independently associated with plasma GCH1 levels (*B* = −7.687, 95% CI: −9.011 to −6.362, *p* < 0.001) (Table 7). In contrast, sex, age, smoking status, hypertension, HDL-C, and LDL-C were not independently associated with GCH1 levels (all *p* > 0.05). These findings are illustrated in the forest plot (Figure 2).

**Table 7:** Multivariable linear regression analysis of factors associated with plasma GCH1 concentration.

Linear model	Unstandardized coefficients		Standardized coefficients	*t*	*p*-value	95.0% CI for *B*		Collinearity statistics	
	*B*	S.E.	Beta			Lower bound	Upper bound	Tolerance	VIF
Constant	15.950	2.075		7.685	0.000	11.850	20.050		
AIS	−7.687	0.670	−0.739	−11.467	<0.001	−9.011	−6.362	0.636	1.572
Sex	−0.962	0.592	−0.092	−1.624	0.106	−2.132	0.208	0.821	1.218
Age	−0.034	0.031	−0.067	−1.122	0.264	−0.095	0.026	0.743	1.346
Smoking	−0.589	0.798	−0.042	−0.738	0.461	−2.166	0.987	0.823	1.215
Hypertension	0.813	0.650	0.079	1.251	0.213	−0.471	2.096	0.666	1.501
HDL-C	−0.769	0.616	−0.072	−1.249	0.214	−1.986	0.447	0.787	1.271
LDL-C	0.489	0.270	0.097	1.815	0.071	−0.043	1.022	0.923	1.083

Multiple linear regression showed that AIS was an independent determinant of the Plasma GCH1 concentration (*B* = −7.687, 95% CI = −9.011 to −6.362, *p* < 0.001). No significant multicollinearity was observed (all VIF < 2; VIF < 10 indicated no severe multicollinearity). B, unstandardized regression coefficient; S.E., standard error; Beta, standardized regression coefficient; CI, Confidence Interval; VIF, variance inflation factor; GCH1, GTP cyclohydrolase 1; AIS, acute ischemic stroke; HDL-C, high-density lipoprotein cholesterol; LDL-C, low-density lipoprotein cholesterol.

### 3.4 Association between GCH1 and neurological deficit severity

**Figure 3:**
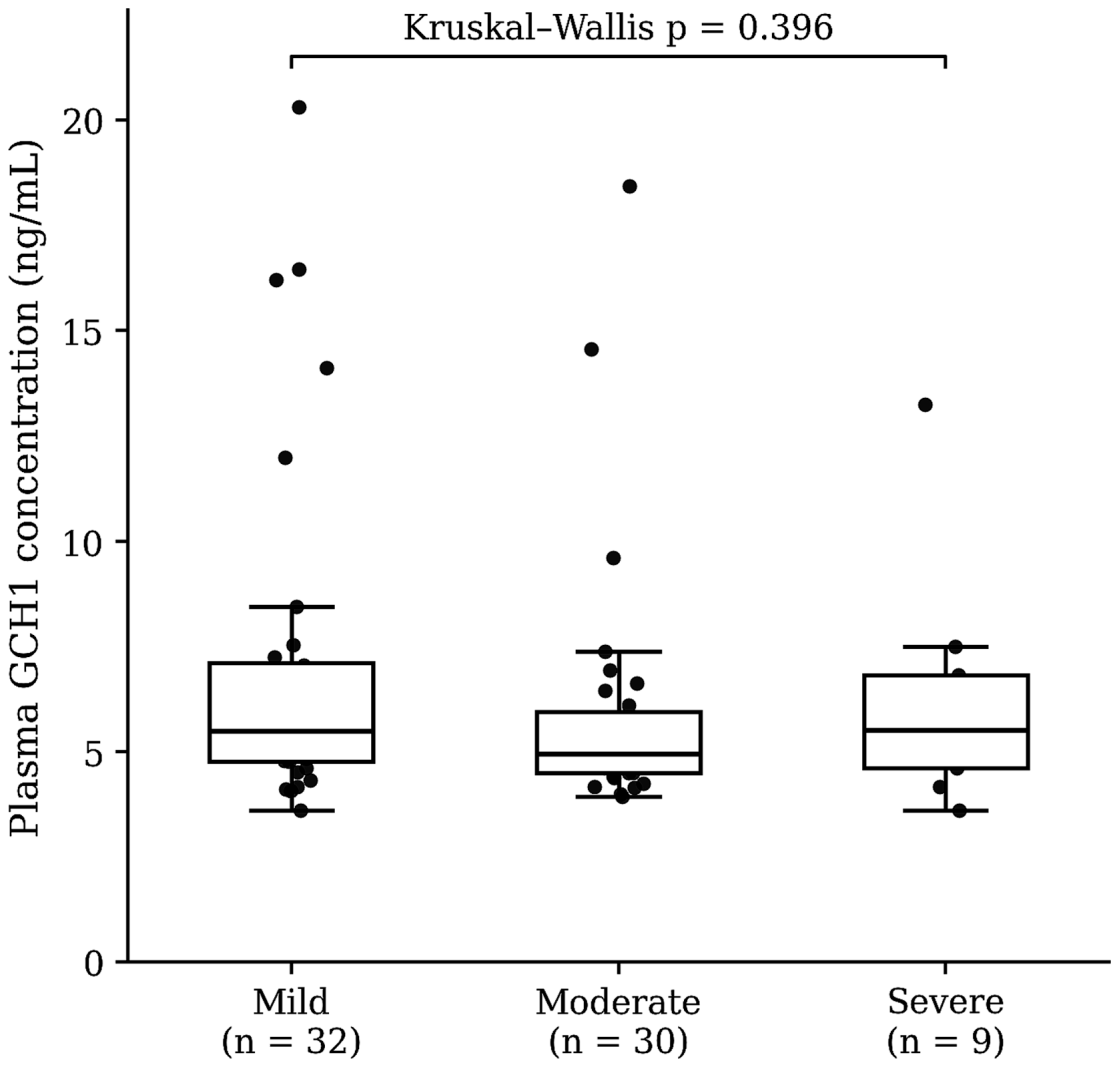
Plasma GCH1 concentrations according to neurological deficit severity in patients with acute ischemic stroke. Patients were classified according to their admission National Institutes of Health Stroke Scale (NIHSS) scores as having mild (NIHSS 0–3; *n* = 32), moderate (NIHSS 4–10; *n* = 30), or severe neurological deficits (NIHSS >10; *n* = 9). Boxes represent the interquartile range (IQR), horizontal lines within the boxes indicate the medians, whiskers extend to 1.5 × IQR, and individual observations are shown as points. Differences among the three groups were evaluated using the Kruskal–Wallis test. No statistically significant difference was observed among the groups (*H*(2) = 1.855, *p* = 0.396).

Although plasma GCH1 concentrations were significantly lower in patients with AIS than in controls (*p* < 0.001; Table 1), no significant correlation was observed between plasma GCH1 concentrations and admission NIHSS scores within the AIS group (Spearman’s rho = −0.034, *p* = 0.778; Table 4). To further examine whether plasma GCH1 concentrations differed according to neurological deficit severity, patients with AIS were classified on the basis of their admission NIHSS scores as having mild (NIHSS 0–3; *n* = 32), moderate (NIHSS 4–10; *n* = 30), or severe neurological deficits (NIHSS >10; *n* = 9). The median plasma GCH1 concentrations were 5.49 ng/mL (IQR, 4.78–7.10 ng/mL) in the mild group, 4.95 ng/mL (IQR, 4.49–5.95 ng/mL) in the moderate group, and 5.50 ng/mL (IQR, 4.62–6.82 ng/mL) in the severe group. Because plasma GCH1 concentrations were not normally distributed, differences among the three severity groups were evaluated using the Kruskal–Wallis test. No statistically significant difference was observed among the groups (H(2) = 1.855, *p* = 0.396; Figure 3). Thus, neither the continuous nor the categorical analysis provided evidence of an association between plasma GCH1 concentrations and neurological deficit severity at admission in this cohort, although the small number of patients in the severe group may have limited the ability to detect between-group differences.

### 3.5 Apparent discriminatory performance of plasma GCH1 for distinguishing AIS cases from controls

**Figure 4:**
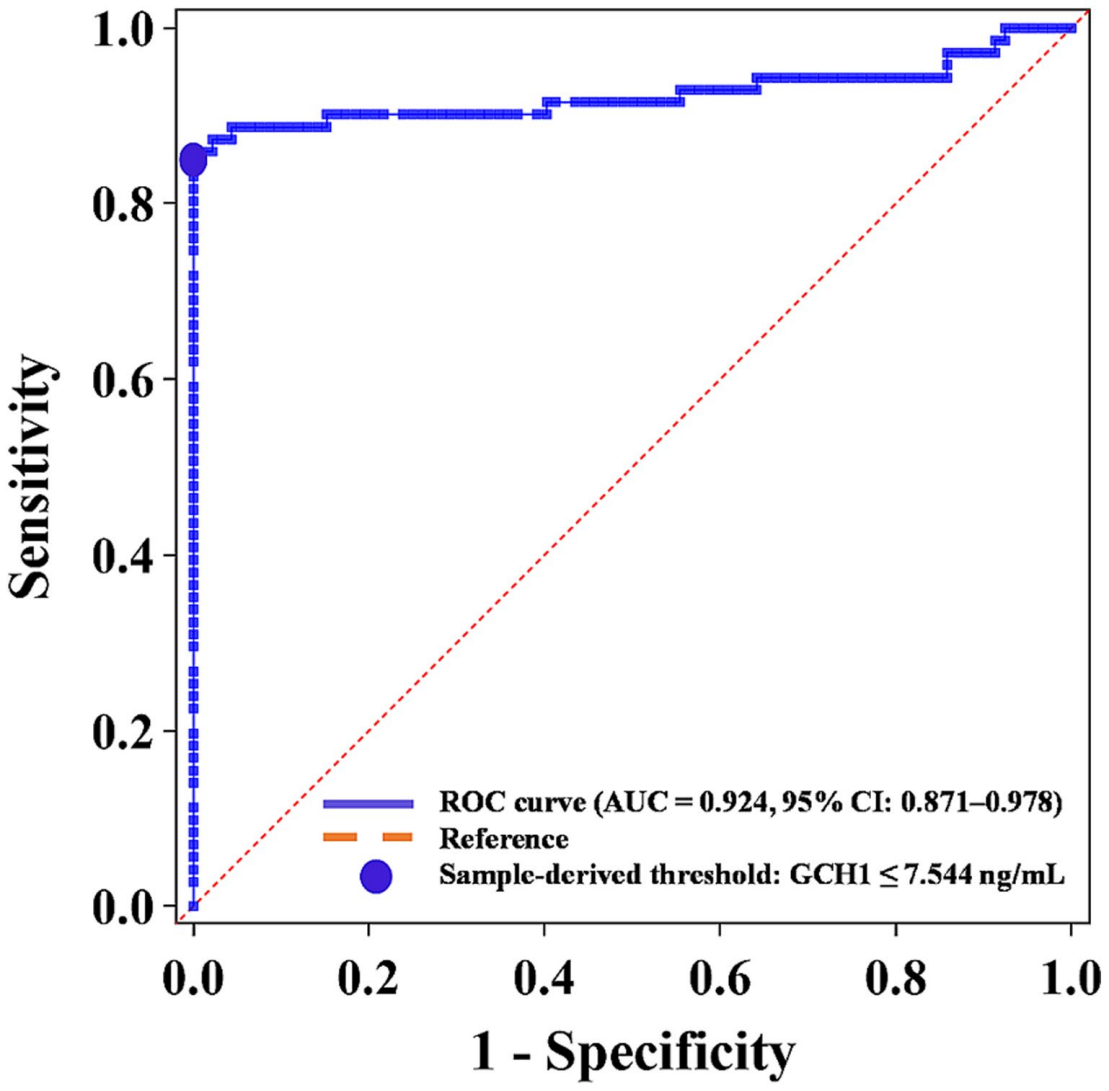
Apparent discriminatory performance of plasma GCH1 for distinguishing AIS cases from controls. The area under the receiver operating characteristic curve was 0.924 (95% CI, 0.871–0.978; *p* < 0.001). The sample-derived threshold determined using the Youden index was ≤7.544 ng/mL, with a sensitivity of 85.9% and a specificity of 100.0% in this case–control sample.

To evaluate the apparent discriminatory performance of plasma GCH1 for discriminating patients with AIS from non-AIS controls within the study cohort, ROC curve analysis was performed in the entire study population (*n* = 163, including 71 AIS patients and 92 controls). Because plasma GCH1 levels were lower in AIS patients, the ROC analysis was performed using −GCH1 as the risk score, with lower GCH1concentrations corresponding to a higher probability of AIS classification. The results are shown in Figure 4 and Table 8.

**Table 8:** Apparent discriminatory performance of plasma GCH1 for distinguishing patients with AIS from controls based on ROC curve analysis.

Parameter	Estimate	95% CI/interval	Description
Total participants	163	—	AIS, *n* = 71; non-AIS controls, *n* = 92; missing GCH1 values, *n* = 0
Direction of analysis	Lower GCH1 indicates AIS	—	ROC analysis was performed using −GCH1 as the risk score
AUC	0.924	0.871–0.978	DeLong 95% CI
Bootstrap AUC	0.924	0.866–0.974	Stratified bootstrap validation with 2,000 iterations
Sample-derived threshold	GCH1 ≤ 7.544 ng/mL	7.381–9.604 ng/mL	Determined by maximizing the Youden index within the study dataset
Sensitivity	85.9%	75.6–93.0%	TP/(TP + FN) = 61/71
Specificity	100.0%	96.1–100.0%	TN/(TN + FP) = 92/92
Youden index	0.859	0.781–0.944	Sensitivity + specificity − 1
Accuracy	93.9%	—	(TP + TN)/*n* = 153/163
Confusion matrix	TP = 61, FP = 0, TN = 92, FN = 10	—	Apparent classification results at the optimal cutoff

ROC, receiver operating characteristic; AUC, area under the ROC curve; AIS, acute ischemic stroke; TP, true positive; FN, false negative; TN, true negative; FP, false positive.

Plasma GCH1 demonstrated strong apparent discrimination between AIS cases and controls, with an area under the ROC curve (AUC) of 0.924 (95% CI, 0.871–0.978; DeLong method). A sample-derived threshold was determined using the Youden index, corresponding to a GCH1 concentration of ≤7.544 ng/mL. At this threshold, the sensitivity was 85.9% (95% CI, 75.6–93.0%) and the specificity was 100.0% (95% CI, 96.1–100.0%) within the present dataset. The corresponding Youden index was 0.859, and the classification accuracy was 93.9%. Based on this threshold, 61 AIS cases were correctly classified, 10 AIS cases were missed, and no non-AIS controls were misclassified as AIS.

Internal validation was conducted using stratified bootstrap resampling with 2,000 iterations. In each bootstrap iteration, the threshold was re-estimated in the bootstrap sample and the discrimination performance was evaluated in the corresponding out-of-bag sample. The internally validated AUC remained high, with a mean value of 0.924 and a 95% percentile interval of 0.866–0.974. The mean validated sensitivity, specificity, and classification accuracy were 0.850, 0.979, and 0.923, respectively. The bootstrap-derived 95% percentile interval for the sample-derived threshold was 7.381–9.604 ng/mL, suggesting reasonable stability of the threshold within this dataset. However, this threshold requires validation in independent, prospective cohorts before it can be considered clinically applicable.

## 4 Discussion

In this study, a marked reduction (> 50%) in the mean plasma GCH1 concentration was observed in AIS patients (6.51 ng/mL) when compared to controls (14.32 ng/mL). Lower GCH1 remained independently associated with AIS after adjustment for age and lipid-related variables. Circulating GCH1 also showed high discrimination between the study groups (AUC, 0.924), although it was not significantly correlated with NIHSS scores. Previous studies have explored the associations of GCH1 gene polymorphisms with ischemic stroke risk and prognosis (22, 23), but the correlation between circulating GCH1 levels and AIS are yet to be reported. Although plasma GCH1 levels were associated with age, smoking status, hypertension, and several lipid-related markers, including TC, APOA1, LDL-C, APOB, Lp(a), and HDL-C, in the overall study population, most of these associations were no longer significant in analyses conducted separately within the AIS and control groups after false discovery rate correction (FDR-adjusted *q* > 0.05), except for a significant association with smoking in the control group. This attenuation suggests that the associations observed in the pooled population may have been driven, at least in part, by differences between patients with AIS and health-examination controls rather than by consistent within-group relationships. Importantly, lower GCH1 remained independently associated with AIS after adjustment for age and lipid-related variables, suggesting that GCH1 dysregulation may represent a distinct feature of AIS pathophysiology. These findings extend existing evidence to the circulating GCH1 level, although the observational design supports association rather than causation.

Endothelial dysfunction and oxidative stress are central contributors to cerebral ischemia (24–26). As a key regulator of tetrahydrobiopterin (BH4) biosynthesis, GCH1 supports antioxidant defense and endothelial nitric oxide synthase (eNOS) coupling (18, 27). Thus, the lower GCH1 levels observed in AIS may be compatible with impaired GCH1/BH4 pathway activity, reduced nitric oxide bioavailability, and increased oxidative stress. This interpretation is consistent with experimental findings that GCH1 downregulation aggravates atherosclerosis (20), whereas GCH1 upregulation improves endothelial function (28). The biological basis of the lower plasma concentration remains uncertain and may involve reduced synthesis, accelerated degradation, or both. Elevated CRP levels and the presence of smoking and hypertension in the AIS group suggest that inflammation and oxidative stress may suppress GCH1 expression or promote protein degradation (29). However, BH4 concentrations, eNOS coupling, nitric oxide bioavailability, and oxidative stress biomarkers were not measured in this study, and circulating GCH1 may not reflect its activity in cerebral endothelium or brain parenchyma. Involvement of the GCH1/BH4 pathway therefore remains biologically plausible but unconfirmed (17).

Beyond its role in NOS coupling, the GCH1/BH4 axis has emerged as an endogenous suppressor of ferroptosis, an iron-dependent form of regulated cell death driven by lipid peroxidation (30, 31). In cancer biology, GCH1 expression is strongly associated with resistance to ferroptosis inducers (32, 33), and ferroptosis has been implicated in neuronal injury after ischemic stroke (34–36). GCH1-mediated BH4 synthesis may limit lipid peroxidation while supporting endothelial function (31, 37, 38); accordingly, reduced GCH1 could be associated with diminished antioxidant protection during AIS. Nevertheless, plasma measurements cannot establish tissue-level ferroptotic activity or a causal role for GCH1. Targeted cellular and animal studies are needed to determine whether GCH1 modulates ischemic injury through ferroptosis-related pathways.

Among lipid-related variables, higher HDL-C was independently associated with lower odds of AIS, whereas higher Lp(a) was associated with greater odds, consistent with previous evidence (39–41). APOA1 mimetic peptides have been reported to upregulate GCH1 and increase BH4 availability, thereby improving endothelial function (19). These observations raise the possibility of an interaction among lipid metabolism, endothelial homeostasis, and GCH1 signaling, although this relationship was not directly evaluated in the present study.

By contrast, TC, LDL-C, and APOB concentrations were lower in the AIS group than in controls. These findings should not be interpreted as contradicting the established role of dyslipidemia in cerebrovascular disease. Sampling occurred during the acute phase, when inflammatory and metabolic responses may transiently reduce circulating lipid concentrations (42). Preadmission lipid-lowering therapy may also have influenced the observed profiles (43). The lower concentrations may therefore reflect acute-phase and treatment-related effects rather than long-term vascular risk.

Although plasma GCH1 concentrations differed significantly between patients with AIS and health-examination controls, they were not significantly correlated with NIHSS scores within the AIS cohort. This pattern suggests that circulating GCH1 may be more closely related to the presence of an AIS-associated pathophysiological state than to the severity of neurological impairment. The absence of a significant association may also reflect limited statistical power due to the moderate sample size and uneven distribution across severity categories. Moreover, NIHSS scores are influenced not only by infarct burden but also by lesion location, vascular territory, and hemispheric laterality (44, 45). Larger studies incorporating quantitative infarct volume, lesion location, vascular territory, laterality, and standardized intervals from symptom onset to blood sampling and neurological assessment are needed to clarify whether GCH1 is associated with specific stroke phenotypes or neurological injury severity.

ROC analysis showed high discrimination between patients with AIS and health-examination controls (AUC, 0.924). At the sample-derived threshold of ≤7.544 ng/mL, GCH1 showed high sensitivity and 100.0% specificity in the study cohort. However, 10 patients with AIS were classified as false negatives, indicating that GCH1 alone cannot reliably exclude AIS. These results support further evaluation of GCH1 as an adjunctive rather than a standalone biomarker. In related cerebrovascular settings, clinical scores and electrocardiographic variables have been investigated for risk stratification and prognosis (46, 47). Future studies should assess whether GCH1 adds value to multimodal models combining neurological findings, neuroimaging, vascular risk factors, electrocardiographic parameters, and other circulating biomarkers.

Several limitations should be acknowledged. First, the cross-sectional case–control design precludes determination of temporal sequence or causality. Because GCH1 was measured after stroke onset, lower concentrations may have preceded AIS or may instead reflect ischemic injury, systemic inflammation, oxidative stress, acute physiological stress, or treatment-related effects; reverse causation cannot be excluded. In addition, GCH1 was measured only once within 72 h of symptom onset, preventing characterization of changes from the hyperacute phase through recovery and limiting evaluation of associations with disease progression or prognosis.

Second, the study used a single-center cohort with a moderate sample size, which may limit generalizability. The sample-derived threshold was selected and evaluated in the same dataset. Although bootstrap resampling provided internal validation, it cannot replace validation in an independent cohort. The observed 100.0% specificity may therefore be optimistic, particularly because the threshold was optimized in a data-driven manner. The proposed sample-derived threshold and performance estimates should be considered preliminary until externally validated in larger multicenter cohorts.

Third, the control group comprised health-examination controls undergoing routine health examinations rather than patients with clinically relevant cerebrovascular or neurological conditions. Although appropriate for exploratory biomarker research, this design may exaggerate between-group discrimination and does not represent real-world differential diagnosis. Future studies should include patients with transient ischemic attack, intracerebral hemorrhage, stroke mimics, and other acute neurological disorders as disease controls.

Fourth, the ROC analysis evaluated GCH1 as an isolated marker and did not assess its incremental performance after accounting for vascular risk factors, stroke subtype, the interval from symptom onset to sampling, treatment status, inflammatory burden, medication exposure, infarct volume, or lesion location. These variables may influence circulating GCH1 concentrations and the apparent discriminatory performance. Standardized sampling, detailed clinical and imaging phenotyping, and multivariable prediction models are needed to determine whether GCH1 adds information beyond established clinical variables or other biomarkers. Accordingly, the present findings are hypothesis-generating and do not establish GCH1 as a clinically useful biomarker for diagnosis, prognosis, severity assessment, or risk stratification. Its clinical utility remains to be established in prospective multicenter studies with independent external validation.

## 5 Conclusion

In this case–control study, plasma GCH1 concentrations were markedly lower in patients with AIS than in health-examination controls, and lower GCH1 remained independently associated with AIS after adjustment for relevant covariates. The attenuation of most associations between GCH1 and demographic, vascular, and lipid-related factors in group-stratified analyses suggests that the relationships observed in the pooled population were partly driven by differences between patients with AIS and health-examination controls. Plasma GCH1 showed strong discrimination between the two groups but was not associated with NIHSS scores, indicating that it may reflect an AIS-associated pathophysiological state rather than the severity of neurological impairment. These findings support GCH1 dysregulation as a potentially distinct feature of AIS and warrant further investigation of the GCH1/BH4 pathway in ischemic cerebrovascular injury. Nevertheless, the cross-sectional, single-center design and lack of external validation preclude causal inference and immediate clinical application. Prospective multicenter studies incorporating longitudinal sampling, clinically relevant disease controls, mechanistic measurements, and independent validation are required to establish the biological significance and adjunctive diagnostic value of circulating GCH1.

## Data Availability

The raw data supporting the conclusions of this article will be made available by the authors, without undue reservation.
